# Self-Exempting Beliefs and Intention to Quit Smoking within a Socially Disadvantaged Australian Sample of Smokers

**DOI:** 10.3390/ijerph13010118

**Published:** 2016-01-11

**Authors:** Ashleigh Guillaumier, Billie Bonevski, Christine Paul, Catherine D’Este, Laura Twyman, Kerrin Palazzi, Christopher Oldmeadow

**Affiliations:** 1 School of Medicine & Public Health, University of Newcastle, P.O. Box 833, Newcastle NSW 2300, Australia; billie.bonevski@newcastle.edu.au (B.B.); laura.twyman@newcastle.edu.au (L.T.); 2 School of Medicine & Public Health, University of Newcastle & Hunter Medical Research Institute, Newcastle 2305, Australia; chris.paul@newcastle.edu.au; 3 National Centre for Epidemiology & Population Health, Australian National University, Canberra 0200, Australia; catherine.deste@anu.edu.au; 4 Clinical Research Design IT and Statistical Support Unit, University of Newcastle & Hunter Medical Research Institute, Newcastle 2305, Australia; kerrin.palazzi@hmri.org.au (K.P.); christoper.oldmeadow@newcastle.edu.au (C.O.)

**Keywords:** self-exempting beliefs, smoking, disadvantage

## Abstract

An investigation of beliefs used to rationalise smoking will have important implications for the content of anti-smoking programs targeted at socioeconomically disadvantaged groups, who show the lowest rates of cessation in the population. This study aimed to assess the types of self-exempting beliefs reported by a sample of socioeconomically disadvantaged smokers, and identify associations between these beliefs and other smoking-related factors with quit intentions. A cross-sectional survey was conducted from March–December 2012 with smokers seeking welfare assistance in New South Wales (NSW), Australia (*n* = 354; response rate 79%). Responses to a 16-item self-exempting beliefs scale and intention to quit, smoker identity, and enjoyment of smoking were assessed. Most participants earned <AUD$400/week (70%), and had not completed secondary schooling (64%). All “jungle” beliefs (normalising the dangers of smoking due to ubiquity of risk) and selected “skeptic” beliefs were endorsed by 25%–47% of the sample, indicating these smokers may not fully understand the extensive risks associated with smoking. Smokers with limited quit intentions held significantly stronger self-exempting beliefs than those contemplating or preparing to quit (all *p* < 0.01). After adjusting for smoking-related variables only “skeptic” beliefs were significantly associated with intention to quit (*p* = 0.02). Some of these beliefs are incorrect and could be addressed in anti-smoking campaigns.

## 1. Introduction

In the U.S. [1], UK [2], Australia [3], Canada [4] and New Zealand [5] general population smoking rates lie between 15%–20%. Comparatively, rates are much higher among those with severe mental illness (32%–90%) [6,7,8,9,10], the homeless (69%–81%) [11,12,13,14], indigenous peoples (32%–42%) [3,5,15], prisoners (50%–74%) [16,17] and those accessing crisis welfare aid (59%) [18]. Consequently, disadvantaged groups experience a disproportionate burden of tobacco-related harm. In order to reduce the disparity in smoking rates, it is vital to improve tobacco control messaging and cessation initiatives among disadvantaged populations.

Despite comprehensive tobacco control measures, many smokers continue to endorse “self-exempting beliefs”, also known as risk-minimizing beliefs or risk denial towards smoking hazards [19,20,21,22]. These beliefs are thoughts used to rationalise or justify continued smoking despite the well-known harms of tobacco use. According to cognitive dissonance theory these beliefs ease the tension arising when one’s knowledge about a behaviour are in conflict with their actions. [23] Four categories of self-exempting beliefs have been identified: (1) “skeptic”—beliefs that discount the harms of smoking; (2) “worth it”—in spite of harms, smoking is worth it; (3) “bulletproof”—the problem does not apply to me; and (4) “jungle”—beliefs that normalise dangers of smoking due to ubiquity of risk [21]. Generally, previous work has suggested that such beliefs are related to quit intentions, and that those from socially disadvantaged backgrounds are more likely to hold self-exempting beliefs [19,20,21,22].

In 1993 a cross-sectional survey of current and former smokers living in low socioeconomic areas of Australia found smokers maintained more self-exempting beliefs than ex-smokers, leading the authors to suggest that becoming an ex-smoker involves a “shedding” of self-exempting beliefs [20]. In 2004 Oakes *et al.* [21] investigated the relationship between self-exempting beliefs and quit intentions among smokers and recent quitters. The cross-sectional study found that, as expected, levels of each of the four self-exempting beliefs scales (“skeptic”, “worth it”, “bulletproof”, “jungle”) decreased across stages of change (*i.e.*, precontemplation, contemplation, preparation) as intention to quit increased. In particular among smokers, “worth-it” beliefs were independent predictors of smokers not planning to quit [21]. Confirming the relationship between rationalizations and quit intentions in a longitudinal study using data from multiple waves of the International Tobacco Control Four-Country project, Borland *et al.* [19] also assessed the predictive strength of these beliefs on quit attempts and quit success. In this study the authors distinguished between self-exempting (“bulletproof”) and risk-minimizing (“jungle”, “skeptic”, “worth it”) beliefs. They found that risk-minimizing beliefs independently predict quit attempts, with those holding the beliefs being less likely to make quit attempts. Neither self-exempting nor risk-minimizing beliefs consistently predicted quit success [19].

Examining how self-exempting beliefs are associated with other factors in socially disadvantaged smokers lives and smoking behaviours may have implications for the content of smoking cessation programs. Factors such as the social context of smoking [24,25,26], enjoyment of smoking [27], and smoker identity [28] are related to quit attempts and success. Identifying erroneous beliefs as well as key rationalisations to target in order to encourage disadvantaged smokers to quit may be helpful in developing better anti-tobacco messaging, cessation services and interventions. This study sought to update and extend the literature by measuring the types of various self-exempting beliefs reported by socioeconomically disadvantaged smokers who were receiving welfare aid from social and community service organisations (SCSOs). In Australia, SCSOs are a highly relevant setting for studying social disadvantage as they represent a setting with a social group who experience diverse and multiple forms of disadvantage such as low income, long-term unemployment, homelessness, substance abuse and mental health issues. The study also aimed to identify associations of self-exempting beliefs, and other smoking-related factors such as enjoyment of smoking and smoker identity, with intentions to quit.

## 2. Experimental Section

### 2.1. Study Design

A cross-sectional touchscreen computer survey was conducted as part of a larger study [29,30] examining smoking in disadvantaged populations between March and December 2012. The complete survey contained 67 questions and took an average of 22.15 min to complete. Only the results relevant to the self-exempting beliefs scale are reported here. The study received approval from the University of Newcastle’s Human Research Ethics Committee (H-2011-0276).

### 2.2. Setting and Sample

Participants were clients of a welfare aid agency in a disadvantaged area of Sydney, in New South Wales (NSW), Australia. Strategies for recruiting hard-to-reach populations were used including the use of a community social service setting. [31] The agency provides emergency relief (*i.e.*, food vouchers, grocery items, financial aid) to members of the community experiencing hardship. Eligible participants were self-reported current smokers attending appointments for the receipt of welfare and financial aid, aged 18 years or over, able to comprehend English and well enough to give informed consent (as judged by agency staff).

### 2.3. Recruitment and Data Collection

The welfare aid agency staff informed clients of an independent research study that was taking place in the service following their emergency relief interview. Interested clients were introduced to a research assistant who explained that the survey was about smoking and tobacco control policies, provided a written information statement and gained informed consent. Participants completed the survey on a touchscreen laptop, with the research assistant available to assist if needed. All participants who completed the survey (*n* = 581) received an AUD$20 grocery card.

Smokers were identified using the survey questions: “Do you currently smoke tobacco products?” with response options “*Yes, daily*”; “*Yes, at least once a week*”; “*Yes, but less often than once a week*” and “*No, not at all*” and “Have you smoked at least 100 cigarettes or a similar amount of tobacco in your life?” (*yes/no/not sure*). Current smokers were classified as those reporting daily smoking, or occasional smoking as well as having smoked 100 cigarettes. Participants reporting not smoking were thanked for their time and shown the end screen.

### 2.4. Measures

#### 2.4.1. Self-Exempting Beliefs

The 16 self-exempting belief items were used from the Oakes *et al.* [21] study. Agreement with each self-exempting belief statement was rated on a scale from 1 to 5 (1 = totally disagree; 2 = disagree; 3 = neither agree nor disagree; 4 = agree; 5 = totally agree). Exploratory factor analysis (EFA) was performed to examine the factor structure of the 16 self-exempting belief questions. The principal components technique with an oblique minimum rotation was used. Rotated factor loadings of >0.4 were considered for inclusion in the factor. The exploratory factor analysis indicated a six factor structure. However, the majority of items loaded on the four pre-defined factors (“skeptic”, “worth it”, “bulletproof”, “jungle”) and accounted for 64.5% of the variance. Confirmatory factor analysis was used to verify the standard four-factor structure (“skeptic”, “worth it”, “bulletproof”, “jungle”) of the 16 self-exempting belief questions. Cronbach’s alpha values were calculated to assess the internal consistency of the four factors; all four factors demonstrated good reliability: “skeptic” (α = 0.83), “bulletproof” (α = 0.83), “worth it” (α = 0.79) and “jungle” (α = 0.74). Confirmatory factor analysis (not reported here) showed that although there was some inconsistency across the range of measures of goodness-of-fit, the fit of the four factor model was reasonable. Therefore the original four factor structure was deemed appropriate and is used throughout the rest of this manuscript.

#### 2.4.2. Smoking Related Measures

Intention to quit was assessed using the item: “Are you seriously thinking of quitting smoking in the next 30 days, the next 6 months, or not at all?” Using the standard “stages of change’ classification for this question, participants were considered as being in stages of *precontemplation* (not at all), *contemplation* (next 6 months) or *preparation* (next 30 days).

Smoker identity was assessed using the categorical item from the UK Smoking Toolkit Study [27]: “Which of these statements most applies to you?” with the listed categories “I hate being a smoker”, “I am unhappy about being a smoker”, “I am happy about being a smoker” and “don’t know/can’t decide”.

Enjoyment of smoking was measured using the item from the UK Smoking Toolkit Study [27]: “How much do you enjoy smoking?” with response options “very much”, “quite a bit”, “not particularly” or “not at all”.

Nicotine dependence was assessed using the Heaviness of Smoking Index [32], which measures time to first cigarette and number of cigarettes per day.

#### 2.4.3. Demographic Characteristics

Age, gender, highest level of education and personal weekly income were assessed.

### 2.5. Statistical Analysis

SAS 9.3 (SAS Institute Inc., Cary, NC, USA) was used for all analyses. Descriptive characteristics of the sample are presented using numbers and percentages for categorical variables and means and standard deviation for continuous variables. Mean belief scores were calculated within each factor (using results from the standard four-factor structure), as the sum of the response scores for each item, scores were then divided by the number of items in the scale to provide a score between 1 and 5. Percentage agreement was measured as percentage of participants who agreed/totally agreed for each question. The independent *t*-test was performed to compare means of belief scores between the three intention to quit groups (precontemplation, contemplation, preparation). As belief scores were similar for the contemplation and preparation quit groups, intention to quit was collapsed into a binary variable for further analysis as described below.

Separate binary logistic regression models were used to assess the association of each of the four self-exempting belief categories (“skeptic”, “worth it”, “bulletproof”, “jungle”) with the outcome of intention to quit smoking within the next 6 months (No/Yes). In addition, smoking related variables (smoker happiness, enjoyment of smoking, nicotine dependence) were adjusted for in a second set of models to ascertain whether each self-exempting belief remained independently associated with quit intention. Age, gender and education were examined as confounders in all models, with the knowledge that in previous studies by Oakes *et al.* [21], sociodemographic differences were found in adherence to self-exempting beliefs; in this study they were not significant in modelling and were therefore not included. Smoking variables included in the model were selected based on content knowledge.

## 3. Results

### 3.1. Sample

During the survey period 738 community service organisation clients were approached to participate and 581 clients completed the survey (79% consent rate). Of the 362 who reported smoking, eight (2%) were excluded for primarily smoking something other than manufactured cigarettes (*n* = 248, 69%) or roll-your-own tobacco (*n* = 106, 29%). The demographic and smoking-related details of the sample (*n* = 354 smokers) are presented in Table 1.

### 3.2. Self-Exempting Beliefs

There was moderate agreement with each of the self-exempting belief statements (see Table 2). “Jungle” beliefs were most commonly reported; one-quarter to half of the sample agreed with each of the individual “jungle” statements. Of the other belief types, approximately 25% of the sample agreed that “*medical evidence on smoking is exaggerated*”, “*more lung cancer is caused by other pollutants than smoking*” and “*the harms of smoking can be overcome with a healthy lifestyle*”.

### 3.3. Relationship Between Self-Exempting Beliefs and Quit Intentions

Table 3 presents the means of the four self-exempting belief factor scores for each of the intention to quit groups (precontemplators, contemplators and preparers). There were statistically significant differences in mean totals between the three quit groups for all four belief factors (*p* < 0.001). As expected, precontemplators held significantly stronger self-exempting beliefs compared to both contemplators (all *p* values < 0.001) and preparers (all *p* values < 0.01). There was no difference in the mean scores for contemplators and preparers for any of the factors, as such quit intention was collapsed into a binary outcome variable: intention to quit within next 6 months (yes = contemplators and preparers; no = precontemplators).

The binary logistic regression presented in Table 4 showed that all four beliefs were associated with intention to quit in the next 6 months in the crude model (all *p*’s < 0.001). However, after adjusting for happiness of smoking, enjoyment of smoking and heaviness of smoking, only the skeptic beliefs were associated with intention to quit in the next 6 months; for each 1 point increase in skeptic belief score, the odds for intending to quit in the next 6 months decreased 33% (OR 0.67, 95%CI: 0.5–0.9; *p* = 0.021).

**Table 1:** Demographic and smoking characteristics of the study sample (*n* = 354).

Characteristic	*n*	%
**Age** (*mean, SD*)	*38*	*10*
**Gender**		
Female	216	61%
**Education**		
Primary School	12	3.4%
High school years 7–10	214	60%
High school years 11–12	51	14%
TAFE/trade qualification	64	18%
University degree	13	3.7%
**Personal weekly income**		
<$200	81	24%
$201–$400	172	52%
>$400	79	24%
**Intention to quit**		
Next 30 days	79	22%
Next 6 months	213	60%
Not at all	62	18%
**Smoker identity**		
I hate being a smoker	130	37%
I am unhappy about being a smoker	128	36%
I am happy about being a smoker	38	11%
Don’t know	58	16%
**Enjoyment of smoking**		
Very much	47	13%
Quite a bit	112	32%
Not particularly	148	42%
Not at all	47	13%
**Nicotine dependence**		
Low	135	39%
Moderate	153	44%
High	61	17%

TAFE, technical and further education.

**Table 2:** Smokers’ agreement with self-exempting belief statements (*N* = 354).

Self-Exempting Belief Statement	Agree/Totally Agree *n* (%)
**Skeptic beliefs**	
Lots of doctors and nurses smoke, so it cannot be all that harmful	33 (9.3%)
The medical evidence that smoking is harmful is exaggerated	86 (24%)
Smoking cannot be all that bad for you because many people who smoke live long lives	44 (12%)
Smoking cannot be all that bad because some top sports people smoke and still perform well	40 (11%)
More lung cancer is caused by such things as air pollution, petrol, and diesel fumes than smoking	79 (22%)
**“Worth it” beliefs**	
I would rather live a shorter life and enjoy it than a longer one where I will be deprived of the pleasure of smoking	41 (12%)
You have got to die of something, so why not enjoy yourself and smoke	49 (14%)
**Bulletproof beliefs**	
Cancer mostly strikes people with negative attitudes	36 (10%)
They will have found cures for cancer and all the other problems smoking causes before I am likely to get any of them	35 (9.9%)
You can overcome the harms of smoking by doing things like eating health food and exercising regularly	82 (23%)
I think I must have the sort of good health or genes that means I can smoke without getting any of the harms	28 (7.9%)
I think I would have to smoke a lot more than I do to put my health at risk	42 (12%)
**Jungle beliefs**	
Everything causes cancer these days	106 (30%)
If smoking was so bad for you, the government would ban tobacco sales	114 (32%)
It is dangerous to walk across the street	167 (47%)
Smoking is not more risky than lots of other things that people do	87 (25%)

**Table 3:** Mean (SD) self-exempting beliefs across quit intention groups.

Belief Factor	Mean (SD) Level of Agreement				*p*-Value ^^^
	Precontemplators	Contemplators	Preparers	Total	
	(*n* = 62)	(*n* = 213)	(*n* = 79)	(*n* = 354)	
	Mean (SD)	Mean (SD)	Mean (SD)	Mean (SD)	
Skeptic	2.5 (1.1)	2.0 (0.9)	1.9 (1.1)	2.1 (1.0)	0.0001
Bulletproof	2.3 (1.0)	1.7 (0.9)	1.8 (1.1)	1.8 (1.0)	0.0003
Worth it	2.5 (1.2)	1.8 (1.1)	1.5 (1.1)	1.8 (1.2)	<0.0001
Jungle	3.3 (1.2)	2.7 (1.1)	2.7 (1.2)	2.8 (1.2)	0.0003

**^^^** *p*-value from ANOVA.

**Table 4:** Logistic regression results showing the association between each self-exempting belief and the intention to quit within the next 6 months; crude, and adjusted for confounders.

Belief	Crude		Adjusted *	
	OR (95%CI)	*p*-Value ^^^	OR (95%CI)	*p*-Value ^^^
Skeptic beliefs	0.59 (0.5–0.8)	<0.001	0.67 (0.5–0.9)	0.0209
Bulletproof beliefs	0.61 (0.5–0.8)	<0.001	0.73 (0.5–1.0)	0.0682
Worth it beliefs	0.59 (0.5–0.7)	<0.001	0.94 (0.7–1.3)	0.6744
Jungle beliefs	0.61 (0.5–0.8)	<0.001	0.77 (0.6–1.1)	0.1227

* Adjusted for happiness of smoking, enjoyment of smoking and heaviness of smoking; **^^^** Wald Chi^2^ *p*-value from binary logistic regression.

## 4. Discussion

This study found that socioeconomically disadvantaged smokers continue to endorse self-exempting beliefs in relation to smoking. In particular, these high priority smokers hold “jungle” and “skeptic” beliefs, which minimise and normalise the dangers of smoking. Consistent with previous studies, these beliefs are related to quit intention, that is, smokers holding any category of self-exempting beliefs were less likely to intend to quit. However, when taking into account other smoking-related variables, in our cohort of disadvantaged smokers it appears that only “skeptic” beliefs are significantly associated with reduced odds of intending to quit.

Clearly it continues to be important to address self-exempting beliefs among current smokers who are socially disadvantaged. Between a quarter to a half of the sample endorsed each of the statements categorised as “jungle” beliefs which may indicate these smokers do not fully understand, or perhaps acknowledge, the extensive risks associated with smoking. Additionally, some of the most highly endorsed beliefs in this study, “skeptic” beliefs, such as “*medical evidence is exaggerated*” and ‘*more cancer is caused by other pollutants*” are incorrect and can be targeted in anti-smoking campaigns. These beliefs, particularly in relation to the exaggeration of medical evidence, have been raised in focus group research with this population of socially disadvantaged smokers [33]. Communication strategies addressing these erroneous and risk-minimising beliefs among disadvantaged smokers should be tested. High emotion, negative health effects messages using personal testimonial or graphic imagery appear to be the most effective way to communicate anti-smoking messaging among low socioeconomic status populations [34,35]. Television advertisements continue to be the most effective channel of delivery for these messages [34]. There is also evidence to suggest that anti-smoking television advertisements that support and complement health warning labels on cigarette packs can increase knowledge, personal relevance of the message and motivation to quit [36].

This study also provides support for the validity of the self-exempting beliefs scale developed by Oakes *et al.* [21] in a current, and disadvantaged, sample. Our factor analysis findings support the classification of common self-exempting belief statements into four scales (“skeptic”, “worth it”, “bulletproof”, “jungle”) among a population of socially disadvantaged smokers. The study also confirms earlier work that these categories of beliefs are related to quit intention. Smokers who held self-exempting beliefs were significantly less likely to have any immediate or future plans to quit in the next 6 months, and this was consistent across all categories of beliefs. Although, after adjusting for other smoking-related variables such as enjoyment and smoker identity, only “skeptic” beliefs remained significantly associated with quit intentions. However, results should also be interpreted with caution as this was a cross-sectional survey and the possibility of reverse causation (*i.e.*, no quit intentions leading to risk-minimising beliefs) exists. Additionally, we are unable to comment on the extent of these beliefs predicting quit attempts and subsequent success, although previous longitudinal research with general population samples suggests risk-minimising and self-exempting beliefs are negatively associated with both intention to quit in the same wave and making a quit attempt at the next wave [19].

Given the persistent social gradient in smoking rates, people in Australia with the lowest socioeconomic status are three times more likely to smoke daily than those in the highest socioeconomic status [3]. It is therefore important to understand the types of self-exempting beliefs that remain prevalent and are associated with quit intentions among this group of smokers in order to identify new anti-smoking messages for future testing. As mentioned above, previous research has indicated that socially disadvantaged smokers are more likely to hold stronger self-exempting beliefs compared with their more advantaged counterparts. The primary strength of this study then, was the current and large sample of highly addicted smokers with socioeconomic barriers to quitting smoking. All participants were presenting for crisis welfare aid and financial assistance at their local community service organisation, had low levels of education and living on or below the Australian poverty line. It should be noted that as attitudes about cessation and smoking may be influenced during times of stress, the recruitment of participants following their service appointment may have biased responses toward quitting as a reduced priority. Additionally, the health literacy of survey participants was not assessed. This sample provides unique information about the self-exempting beliefs of a priority population.

## 5. Conclusions

In conclusion, socioeconomically disadvantaged smokers continue to hold self-exempting beliefs that serve to minimise the risks of smoking. In particular, among these smokers beliefs that discount the harms of smoking (“skeptic” beliefs) appear to be associated with reduced quit intentions. It continues to be important for tobacco control initiatives to address erroneous beliefs about the harms of smoking, particularly among those with very high smoking rates.
